# Comparative Transcriptome and DNA methylation analyses of the molecular mechanisms underlying skin color variations in Crucian carp (*Carassius carassius L.*)

**DOI:** 10.1186/s12863-017-0564-9

**Published:** 2017-11-09

**Authors:** Yongqin Zhang, Jinhui Liu, Wen Fu, Wenting Xu, Huiqin Zhang, Shujuan Chen, Wenbin Liu, Liangyue Peng, Yamei Xiao

**Affiliations:** 1 State Key Laboratory of Developmental Biology of Freshwater Fish, Hunan Normal University, Changsha, Hunan 410081 China; 2 School of Life Sciences, Hunan Normal University, Changsha, Hunan 410081 China

**Keywords:** Body color, Pigment cell, Transcriptome, DNA methylation, Crucian carp

## Abstract

**Background:**

Crucian carp is a popular ornamental strain in Asia with variants in body color. To further explore the genetic mechanisms underlying gray and red body color formation in crucian carp, the skin transcriptomes and partial DNA methylation sites were obtained from red crucian carp (RCC) and white crucian carp (WCC). Here, we show significant differences in mRNA expression and DNA methylation sites between skin tissues of RCC and WCC.

**Results:**

Totals of 3434 and 3683 unigenes had significantly lower and higher expression in WCC, respectively, compared with unigenes expressed in RCC. Some potential genes for body color development were further identified by quantitative polymerase chain reaction, such as mitfa, tyr, tyrp1, and dct, which were down-regulated, and foxd3, hpda, ptps, and gch1, which were up-regulated. A KEGG pathway analysis indicated that the differentially expressed genes were mainly related to mitogen activated protein kinase (MAPK), Wnt, cell cycle, and endocytosis signaling pathways, as well as variations in melanogenesis in crucian carp. In addition, some differentially expressed DNA methylation site genes were related to pigmentation, including mitfa, tyr, dct, foxd3, and hpda. The differentially expressed DNA methylation sites were mainly involved in signaling pathways, including MAPK, cAMP, endocytosis, melanogenesis, and Hippo.

**Conclusions:**

Our study provides the results of comparative transcriptome and DNA methylation analyses between RCC and WCC skin tissues and reveals that the molecular mechanism of body color variation in crucian carp is strongly related to disruptions in gene expression and DNA methylation during pigmentation.

**Electronic supplementary material:**

The online version of this article (10.1186/s12863-017-0564-9) contains supplementary material, which is available to authorized users.

## Background

Fish display numerous colors and color combinations. Some species have been used as ornamental fish, in which body color and pattern are important characteristics. The crucian carp (*Carassius auratus* L.) is a freshwater fish in the family Cyprinidae (order Cypriniformes) and is one of the most commonly kept aquarium fish [1]. As a colored fish with variants in body color, the crucian carp is a popular ornamental strain, as they are produced in a variety of colors including red, orange/gold, yellow, white, and brown, or black. Red crucian carp (RCC; *C. auratus* red var.), or the common goldfish, is one of the earliest domesticated fish and has become a popular ornamental fish owing to its red/orange body color. White crucian carp (WCC; *C. auratus cuvieri* Temminck et Schlegel) is back-gray in body color [2, 3].

Pigment cells on the body surface provide body color pigment patterns and are usually classified according to pigment composition. Fish body color is controlled by the distribution of pigment cells, such as melanocytes, xanthophores, erythrophores, and iridocytes [4–6]. Melanocytes, contain a large number of melanin granules and are able to absorb the specific wavelengths of incident light that make fish appear grey-black. Xanthophores and erythrophores hold carotenoids and pteridines and provide the yellow, orange and red colors of fish.

The genetic control rules for body color formation in RCC may be differ from those in WCC. Actually, melanocytes appear during the RCC embryo and larval stages. Then, the body color of RCC changes from dark-gray to red-orange as the melanocytes disappear and body color begins to form [7]. It has been suggested that body color formation in RCC is subject to complex controls by multiple agents not only via the pigment biosynthetic pathway and chromatophore differentiation pathway but also the autophagy and apoptosis pathways [8]. DNA methylation is an important epigenetic modification of the eukaryotic genome and plays an important role maintaining the biological functions of higher organisms, such as normal cellular functions, genetic markers, embryonic development, aging, and human tumorigenesis [9, 10]. Li (2015) performed the DNA methylation analyses for red skin and white skin of koi carp, there showed that the DNA methylation levels of two selected DEGs inversely correlated with gene expression, indicating the participation of DNA methylation in the coloration [11]. In this study, we chose the methylation-RAD detection method to further explore the mechanisms underlying gray and red body color formation in crucian carp. We compared the skin tissues of RCC and WCC using transcriptome sequencing and methylation-RAD sequencing analyses (simple genomic methylation site detection method) [12, 13]. We analyzed the differentially expressed genes (DEGs) and different DNA methylation levels using the transcriptome and MethylRAD data. This study attempts to reveal the molecular and genetic mechanisms as well as epigenetic modification of coloration formation in two kinds of crucian carp.

## Results

### Transcriptome assembly and annotation

After filtering low quality and short sequences, we obtained about 54 and 52 million clean reads in the skin tissues of RCC and WCC, respectively. The complete clean reads for these libraries have been uploaded onto the NCBI Sequence Read Archive site SRS2441209, SRX3105778, SRR5947250, and SRS2441217. After eliminating redundant sequences and filtering short sequence (bp ≤ 400), 56,564 and 56,612 assembled transcripts were obtained from the skin tissues of RCC and WCC, respectively. Average read size, Q20 percentage, and other Parameters are presented in Table 1.

**Table 1:** Overview of sequencing and assembly

Sample	RCC	WCC
Total Raw Reads	58,450,968	55,645,610
Total Clean Reads	54,626,008	52,222,670
Total Clean Nucleotides	4,916,340,720	4,700,040,300
Q20 percentage	97.95%	98.01%
N percentage	0.01%	0.01%
GC percentage	47.10%	47.27%
N50	1038	1074
Mean	597	602
Unigenes	56,564	56,612

### Functional enrichment analysis of annotated Unigenes

**Fig. 1:**
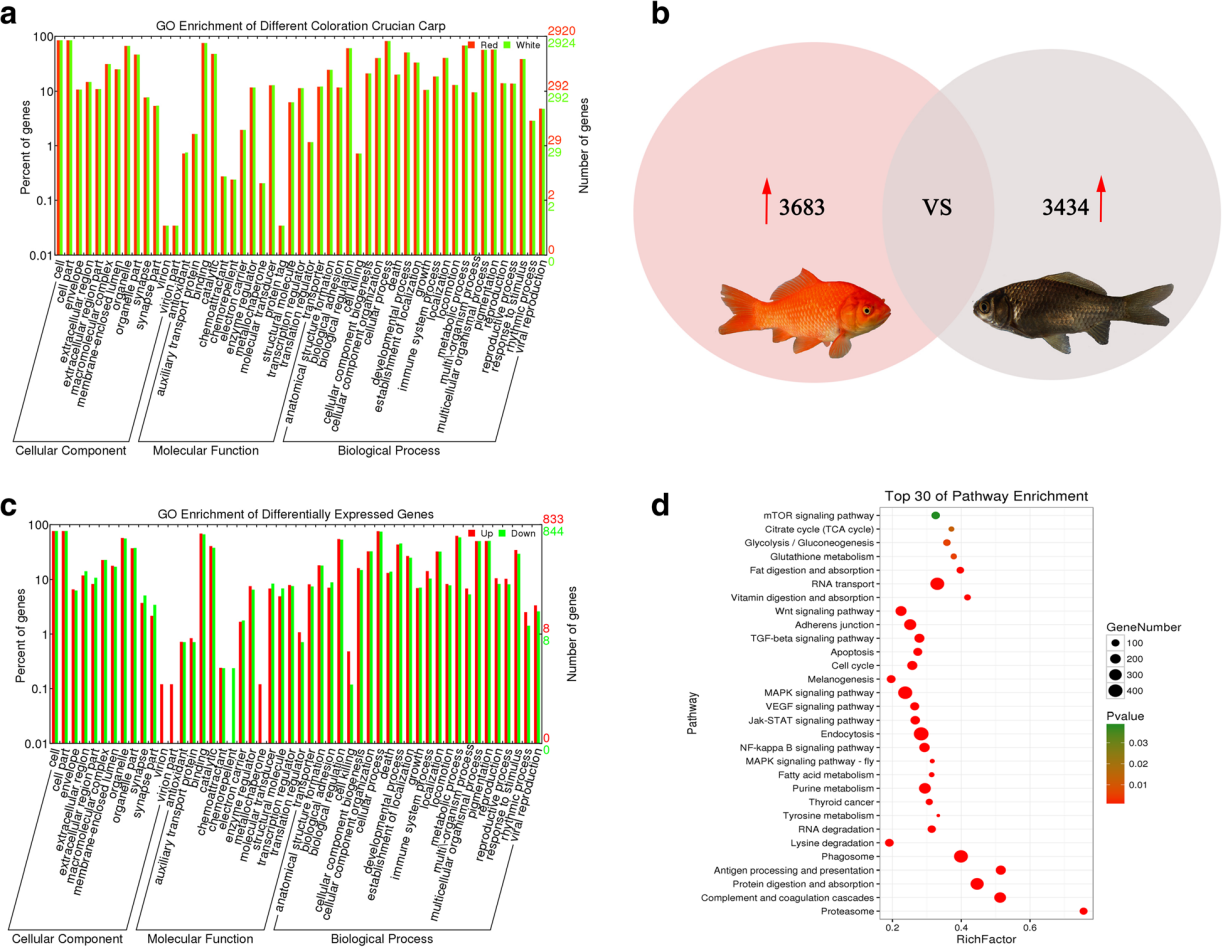
Summary of GO and KEGG pathway enrichment analysis. **a** Gene ontology classification of unigenes between the RCC and the WCC. “Red” represents the unigenes that are expressed in the RCC; “White” represents the unigenes that have expression inthe WCC. **b** Differentially gene expression in the skin of the RCC and the WCC. **c** Gene ontology classification of differentially expressed unigenes between the RCC and the WCC. “Up” represents the unigenes that are over-expressed in the RCC; “Down” represents the unigenes that have reduced expression in the WCC. **d** KEGG enrichment top30 bubble chart. The x-axis Enrichment Score is an enrichment score. The more the number of different genes is the larger bubble. The bubble color is changed by red-green, and its enrichment *p*-value is large

 Twenty four thousand nine hundred seventy one unigenes were obtained from the crucian carp skin, which annotated with Gene Ontology (GO). The GO term classifications were similar between RCC and WCC, with little variation in molecular function (Fig. 1a). Using COG annotation, 16,804 unigenes in the crucian carp group were assigned into 25 functional categories. A total of 34,193 annotated sequences were enriched into 258 predicted KEGG pathways in the transcriptome from the skin of crucian carp. Detailed annotated information is given in Additional file 1: Table S1.

### Comparative and enrichment analysis of differentially expressed genes

Using the MARS method [14], we defined an expression level of >2-fold changes and false discovery rates (FDR) of ≤0.001 to filter genes with significant differential expression between the skin tissues of RCC and WCC. A total of 7117 genes were differentially expressed between RCC and WCC. Overall, 3683 were up-regulated in RCC and 3434 were down-regulated compared with their levels in WCC (Fig. 1b). The DEGs of RCC compared to those of WCC were mainly in the cellular component, molecular function, and biological process categories (Fig. 1c). And the analysis of KEGG pathway revealed that multiple pathways, including the “MAPK signaling pathway”, “Wnt signaling pathway”, “cell cycle”, “VEGF signaling pathway”, “endocytosis”, “melanogenesis” and “tyrosine metabolism” were clearly enriched in the crucian carp DEGs (Fig. 1d).

### Analysis of some pigmentation related expressed genes and RNA-Seq data validation

**Fig. 2:**
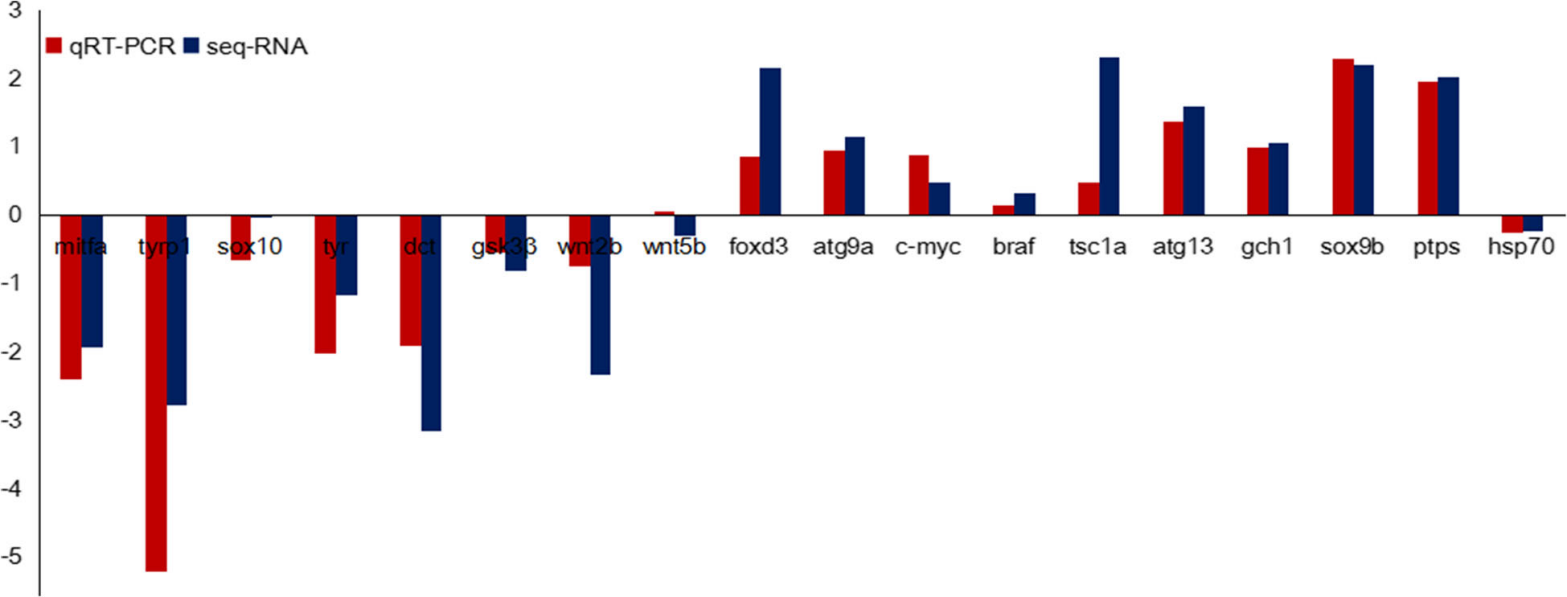
Comparison of relative fold change of Illumina sequencing with qRT-PCR results between the RCC skin and the WCC skin

To examine the reliability of the RNA-seq results, eight DEGs (tyr, mitfa, tyrp1, dct, foxd3, hpda, gch1, and ptps) involved in the development of body color were selected for validation using qPCR. As shown in Fig. 2, the qRT-PCR expression patterns of the eight DEGs were in agreement with the RNA-seq data. A number of DEGs related to the occurrence of pigment cells were selected.

**Fig. 3:**
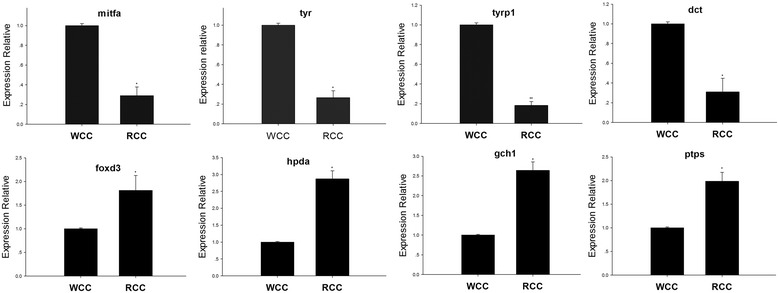
Real-time PCR analysis for a part of the differentially expressed genes. The lowercase letters show the significant differences (*P < 0.05) (means ± SD of relative expression; *n* = 3 for each group)

As shown in Table 2 and Fig. 3, the mRNA expression levels of key genes for tyrosine synthesis, such as those encoding MITFa, TYR, tyrosinase-related protein 1 (TYRP1) and DCT, were all down-regulated in RCC skin compared to that in WCC skin. Furthermore, the mRNA expression levels of foxd3, hpda, ptps, and gch1 were up-regulated in skin tissues from RCC compared to those in WCC.

**Table 2:** Analysis of differentially expressed genes related to pigmentation

Gene	RCC_FPKM	WCC_FPKM	*P*_value	Note
Wnt2b	1.112	4.019	3.00E-50	Wnt family member 2b
Pax3a	0.853	8.546	4.00E-18	Paired box protein Pax-3a
Mitfa	0.647	2.645	1.00E-59	Microphthalmia associated transcription factor a
Foxd3	10.032	1.257	0	Forkhead box D3
Tyr	1.094	2.456	0	Tyrosinase
Tyrp1b	0.435	2.992	0	Tyrosinase-related protein 1b
Dct	0.214	2.124	2.00E-92	Dopachrome tautomerase
Silva	0.684	3.794	2.00E-113	Silver protien a
Hpda	103.485	37.312	1.00E-139	4-hydroxyphenylpyruvate dioxygenase a
Mc1r	0.863	2.111	2.00E-27	Melanocortin receptor 1
Hgd	14.715	5.784	2.00E-87	Homogentisate1,2-dioxygenase
Gch1	4.461	2.152	7.00E-23	GTP cyclohydrolase 1
Xdh	6.877	2.571	0	Xanthine dehydrogenase/oxidase
Kitla	5.989	2.667	0	Kit ligand a
Ptps	6.028	0.547	6.00E-120	6-pyruvoyl tetrahydropterin synthase
Atg4a	1.311	0.214	0	Autophagy related 4 homolog A
Atg9a	9.862	4.478	0	Autophagy related 9 homolog A
Tfe3a	9.802	4.732	1.00E-23	Transcription factor binding to IGHM enhancer 3a
C-fos	7.477	63.901	7.00E-99	C-Fos protein
Gnas	2.457	5.253	1.00E-07	Guanine nucleotide-binding protein G(s) alpha
Gch2	1.194	9.245	5.00E-172	GTP cyclohydrolase 2
Tp53	3.013	1.717	0	Tumor protein p53
Chuk	3.993	8.183	1E-136	Inhibitor of nuclear factor kappa-B kinase alpha
Il3ra	5.661	0.692	7.00E-14	Interleukin 3 receptor alpha
Il1r1	7.693	1.916	3.00E-177	Interleukin 1 receptor type I
Il-8	13.218	6.084	2.00E-26	Interleukin-8
C-fms	6.907	1.148	0	Colony stimulating factor 1 receptor
Pax6b	3.518	0.922	1.00E-50	Paired box protein Pax-6b
Tsc1a	14.976	3.016	0	Tuberous sclerosis 1a
Sox9b	4.202	2.215	9.00E-169	HMG-box transcription factor Sox9b
Erk2	2.078	4.178	0	Extracellular signal regulated protein kinase 2

### Analysis of DNA methylation site distribution and DNA methylation levels

**Fig. 4:**
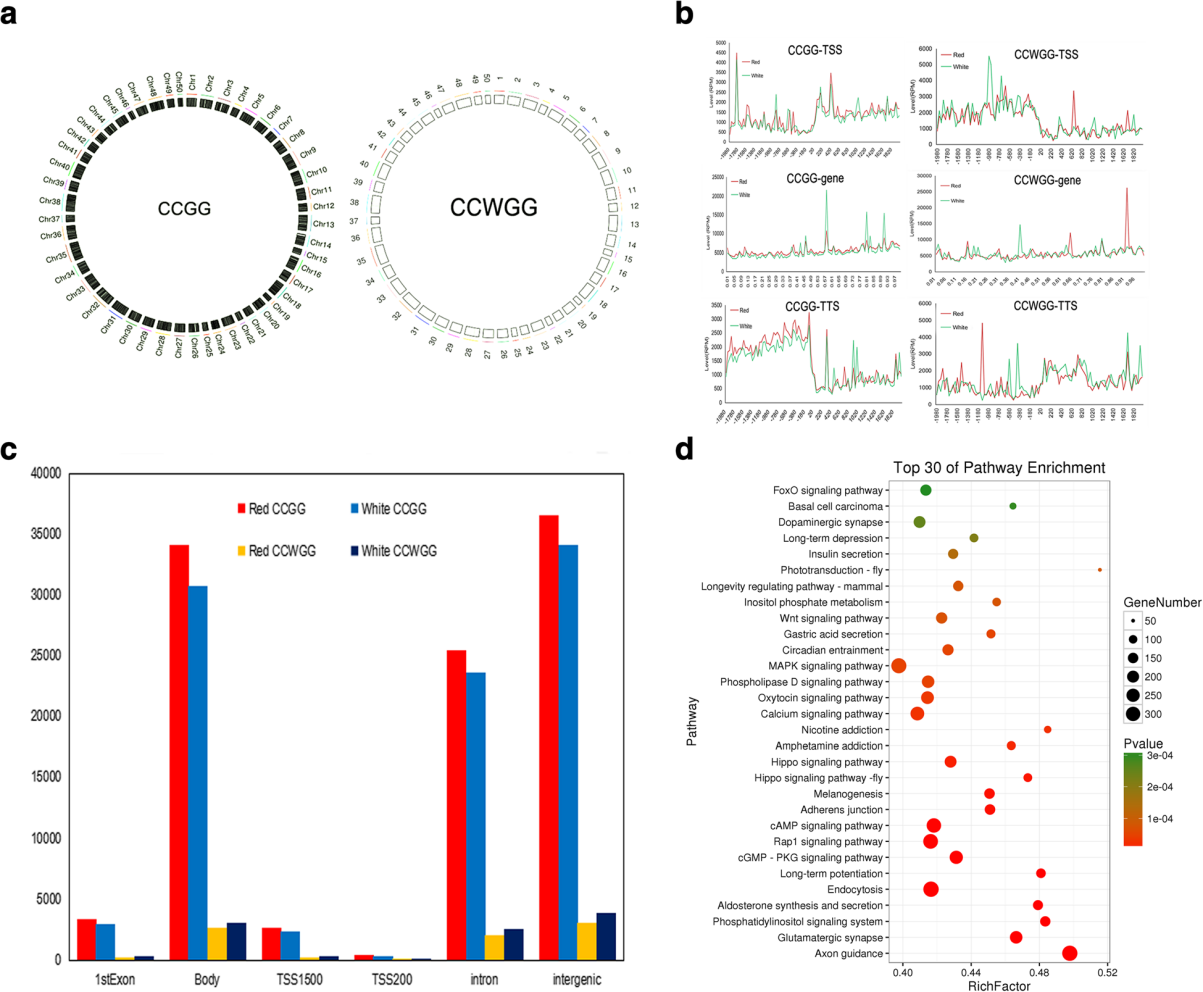
DNA methylation site on gene DNA methylation levels and function components distribution. **a** The distribution of differential DNA methylation sites on chromosomes. **b** DNA methylation level change line chart. The red curve represents the methylation level of the RCC, and the green curve represents the methylation level of the WCC. **c** DNA methylation site on different gene function components distribution histogram. 1stexon: first exon; Body: gene area; TSS200: gene starting sites within 200 bp upstream area; TSS1500: gene starting sites within 1500 bp upstream area; Exon: intron area; Intergenic: intergenic region. **d** KEGG enrichment analysis DNA methylation gene on top30 bubble chart. The x-axis Enrichment Score is an enrichment score. The more the number of different genes is the larger bubble. The bubble color is changed by red-blue-green-yellow, and its enrichment *p*-value is large

The MethylRAD analysis was used to characterize the cytosine methylation pattern in the skin tissues of RCC and WCC. The high-quality methylation tag libraries of the two samples were 18.08–21.45% comparable to unique positions in the genome (Additional file 1: Table S2). The sequencing depths of the DNA methylation sites (CCGG sites and CCWGG sites) in each sample are shown in Table 3. Totals of 96,122 and 7961, CCGG and CCWGG DNA methylation sites were found in RCC, with average methylation coverage of 30.71 and 9.18, respectively. Totals of 88,536 and 9534 CCGG and CCWGG DNA methylation sites were found in WCC with average methylation coverage of 28.35 and 9.02, respectively. These results show that the DNA methylation level of RCC was slightly higher than that of WCC (Table 3). The RCC and WCC differential methylation sites (CCGG/CCWGG) were identified on the chromosomes of common carp (Fig. 4a). We obtained the DNA methylation site annotation of the common carp genome, and found that the DNA methylation site distribution curve had TSS representing an upstream sequence centered on the transcription initiation site, and TTS representing a downstream sequence centered on the transcription termination site (Fig. 4b). We also discovered that these DNA methylation sites were mainly distributed in the gene body, intron, and intergenic areas, and that there were slightly more DNA methylation sites in RCC than that in WCC (Fig. 4c).

**Table 3:** DNA methylation site coverage depth in two coloration crucian carp skins

Sample	CCGG		CCWGG	
	Site	Depth	Site	Depth
WCC	88,536	28.35	9534	9.02
RCC	96,122	30.71	7961	9.18
Average	92,329	29.53	8748	9.10

### Enrichment analysis of differential methylation site-related genes

The function of a gene was described by a GO function enrichment analysis of the gene where the differential DNA methylation sites were located (Note Download link: https://ftp.ncbi.nlm.nih.gov/genomes/all/GCF/000/951/615/GCF_000951615.1_common_carp_genome/GCF_000951615.1_common_carp_genome_genomic.gff.gz). The GO enrichment analysis top30 bar graph is shown in Additional file 1: Fig. S1. The “MAPK signaling pathway”, “cAMP signaling pathway”, “Hippo signaling pathway-fly”, “Endocytosis”, “Melanogenesis” and “Hippo signaling pathway” were significantly enriched in crucian carp. Some of these pathways are associated with pigmentation synthesis (Fig. 4d).

### Partial differential expression DNA methylation site gene analysis

**Fig. 5:**
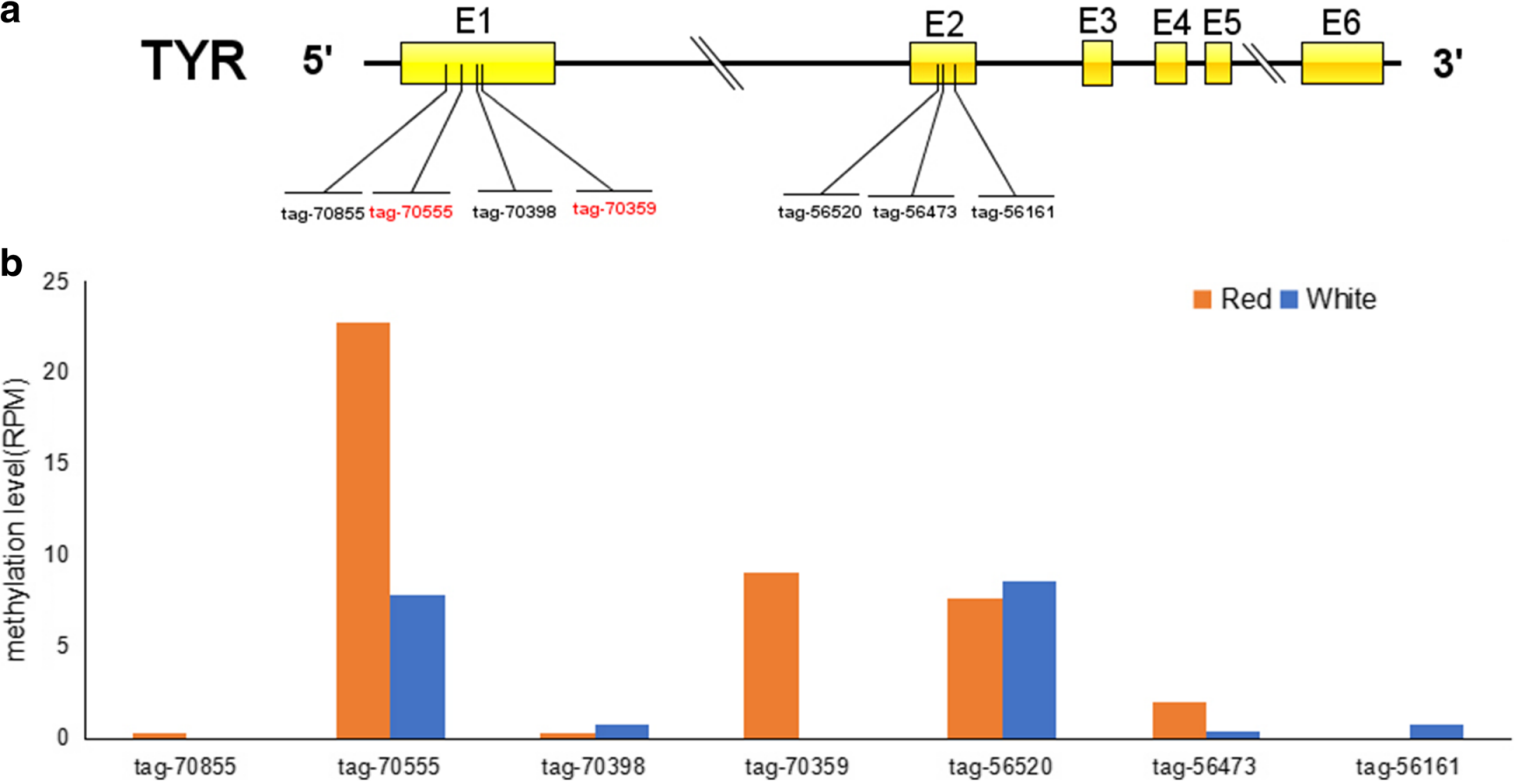
The tyrosine genomic DNA methylation site analysis. **a** The gene structure of TYR and its associated MethylRAD tags. The differentially DNA methylated sites are indicated by red tag names. **b** Comparison of DNA methylation levels between two groups for each tag in the tyr gene

Genes with the same trends of change in expression levels were determined between the RNA-seq and MethylRAD data (Table 4). We obtained the DNA methylation site distribution of the TYR gene that is closely related to melanin synthesis (Fig. 5a). Figure 5b shows a comparison of the DNA methylation site coverage level for each tag in the TYR gene between RCC and WCC.

**Table 4:** Analysis of differentially expressed genes and their DNA methylation levels

Gene ID	Red-FPKM	White-FPKM	Log2 (R/W)	Red-RPM	White-RPM	Log2 (R/W)
Tyr	1.09	2.46	−1.16	32.46	8.63	1.91
Mitfa	0.65	2.85	−1.92	31.13	11.38	1.45
Foxd3	10.03	2.257	4.44	0.33	16.87	−5.67
Dct	0.21	2.12	−3.31	9.72	3.53	1.46
Hpda	103.48	37.31	1.47	1.26	23.45	−3.86−4.21

### Protein expression of three major pigment genes in skin tissues of Crucian carp

**Fig. 6:**
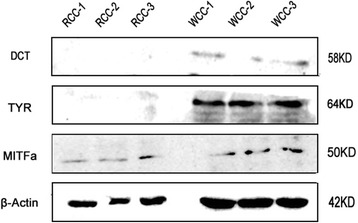
Verification of the expression of MITFa, TYR and DCT protein by western blot analysis. The “RCC-1”, “RCC-2,” and “RCC-3” represent the bands from the RCC; “WCC-1”, “WCC-2,” and “WCC-3” represent the bands from the WCC

A western blotting analysis with commercially available antibodies was performed to determine whether the DEG findings were reflected at the protein level. The relative expression levels of MITFa, TYR, and DCT were higher in WCC skin tissues than those of RCC (Fig. 6), which was consistent with the results of the transcriptome mRNA differential expression analysis, and partially supports the reliability of mRNA differential expression analysis.

## Discussion

DNA methylation is essential for normal development and is associated with a number of key processes, including genomic imprinting, X-chromosome inactivation, repression of transposable elements, aging, and carcinogenesis [15, 16]. In this study, we constructed RNA-seq and DNA methylation libraries from skin tissues of RCC and WCC using transcriptome sequencing and the MethylRAD methods and discovered some DEGs and differentially methylated genes associated with pigmentation. There were 7117 DEGs found between RCC and WCC. A GO enrichment analysis of the DEGs revealed that the variation in pigmentation was related to cellular components and biological processes, such as pigmentation, biological regulation, and cellular process terms. Meanwhile, we found that some differentially methylated genes are enriched in some signaling pathways, including MAPK signaling pathway, cAMP signaling pathway, Wnt signaling pathway and Melanogenesis. This suggests that DNA methylation may be related to the formation of body color in crucian carp.

Melanocytes contain melanin, which is mainly synthesized from tyrosine. The three important enzymes were involved in melanin synthesis, including tyrosinases (TYR), tyrosinase-related protein 1 (TYRP1) and dopachrome tautomerase (DCT or TYRP2). Tyrosinase (TYR) carries out tyrosine hydroxylation to L-DOPA, which is rapidly oxidized to DOPAquinone. The DOPAquinone spontaneously undergoes cyclization to DOPAchrome. Under the action of the dopachrome tautomerase (DCT or TYRP2) and tyrosinase-related protein 1 (TYRP1), the DOPAchrome form DHICA-melanin is insoluble, which rapidly oxidizes and polymerizes to form dark or brown-black pigments. Our results show that the mRNA and protein expression levels of tyr and dct in RCC were significantly lower than those in WCC, and that the tyr and dct gene have modified DNA methylation sites, which were negatively correlated with their mRNA expression levels. This suggests that the tyrosine metabolism pathway was associated with different skin color in red crucian carp and white crucian carp. Besides, we found that the mitfa mRNA and protein level in skin tissue of RCC was significantly lower than that of WCC, and DNA methylation level was negatively correlated with gene expression level. The mitfa gene is a key factor regulating survival and development of melanocytes. Mitfa directly regulates the expression of multiple genes necessary for melanophore development, including dct, tyr, tyrp1, c-kit, and bcl2 [17]. Ectopic mis-expression of mitf is sufficient to confer a melanoblast phenotype [18, 19]. Some transcription factors can act on the promoter region of MITF, thereby promoting the expression of MITF, such as beta-catenin, Paired box 3 (PAX3), SRY-box containing gene10 (SOX10) [20]. In zebrafish, null mutations in the microphthalmia-associated transcription factor (MITF) ortholog mitfa lack all neural crest-derived melanocytes but retain the other two neural crest pigment cell types, xanthophores and iridophores [21]. This suggests that mitfa may be play important roles information of body color in crucian carp.

We also discovered some differentially DNA methylated genes involved in the MAPK and cAMP signaling pathways, which might be related to pigmentation in crucian carp. Extracellular signal regulated kinase 2 (ERK2)/MAPKs are reported to play an important role during post-translational modification of mitf [22, 23]. Activation of c-KIT in melanocytes results in phosphorylation of MITF at Ser73 by ERK2. Phosphorylation at Ser73 increases recruitment of transcriptional coactivator p300 [24], while simultaneously targeting MITF for ubiquitin dependent proteolysis [25]. Our results indicate that mRNA expression of the erk2 gene was lower in RCC than that in WCC, leading to the inability of mitf to be phosphorylated and move to the nucleus, which may have decreased mitf mRNA level. This could also affect the expression of some phosphorylation genes, such as cell cycle and apoptosis-related genes. In the cAMP signaling pathways, Alpha-melanocyte stimulating hormone (a-MSH) is one such peptide hormone that has been suggested to modulate the pigment response. Studies have shown a role of a-MSH in promoting pigment synthesis a-MSH binds to the melanocortin 1 receptor (MC1r), which is a member of the seven-transmembrane receptor family that activates adenylcyclase via G-protein signaling [26]. Although cAMP triggers numerous downstream effects, one important target is the MITF gene, which is transcriptionally up-regulated by cAMP signaling in a melanocyte-restricted fashion, thus linking extracellular signals to MITF expression and the transcriptional regulation of pigmentation [27]. Meanwhile, cAMP may be a second messenger in the dispersion of pigment induced by prolactin and that a novel protein receptor coupled with a Gs protein may be present in the membrane of erythrophores and xanthophores of teleost fish [28]. In this study, we found that the mRNA level of MC1R was down-regulate in the skin from RCC to WCC. In summary, DNA methylation may be involved in the formation and development of different body color patterns in crucian carp.

In addition, we found that 4-hydroxyphenylpyruvate dioxygenase (HPDA) in the tyrosine metabolic pathway is highly expressed in the skin of RCC, which is the key enzyme in tyrosine metabolism. HPDA catalyzes conversion of hydroxyphenyl pyruvate to ursolic acid (HGA), and HGA enters the TCA cycle through a series of reactions [29]. On the other hand, HPDA reduces blood tyrosine level, thereby reducing the source of melanin [30]. Furthermore, foxd3 mRNA levels in WCC were significantly higher thanthose in RCC. The forkhead transcription factor, Foxd3, represses expression of Mitf, and its negative regulation is also important for the cell fate specification [31–34]. The DNA methylation level of the Foxd3 gene was higher in WCC than that in RCC, and negatively correlated with its mRNA level. Foxd3 represses expression of mitfa in red and white koi, which not only inhibits the expression of melanocytes but also contributes to the formation of xanthophores and erythrophores [35]. In addition, foxd3 interacts with pax3a to activate the MITF promoter [36] and might respond to interleukin (IL) signaling via a poorly understood pathway [37]. Our results indicate that the pax3a mRNA level was reduced in RCC compared to that in WCC. The mRNA levels of IL3 and IL8 were higher in RCC than those in WCC. These results indicate that foxd3 reduces the formation of melanocytes and may increase the formation of xanthophores and erythrophores. Therefore, the high foxd3 mRNA level in RCC may be associated with the different coloration patterns established in crucian carp.

In addition, the BH4 de novo biosynthetic pathway has been associated with the synthesis of pteridine pigments in goldfish and zebrafish [38]. Three enzymes are involved in this pathway, such as GTP cyclohydrolase I (EC 3.5.4.16; GCH), 6-pyruvoyl tetrahydropterin synthase (EC4.6.1.10; PTPS), and sepiapterin reductase (EC 1.1.1.153). These enzymes catalyze the conversion of GTP to dihydroneopterin triphosphate, dihydroneopterin triphosphate to 6-pyruvoyl tetrahydropterin, and 6-pyruvoyl tetrahydropterin to BH4, respectively [39]. The gch1 and ptps mRNA expression levels were significantly upregulated in RCC compared to those in WCC. This includes introduction of pteridine signaling pathways in the xanthophores and erythrophores of RCC.

## Conclusions

We evaluated the global gene expression patterns and DNA methylation patterns in the skin of crucian carp with different coloration patterns. Functional analyses of differentially expressed transcripts revealed that the molecular mechanism of body coloration in crucian carp is strongly related to disruptions in gene expression during pigmentation. Specifically, the expression of genes is associated with different coloration. Our results provide a foundation for further characterization of gene expression during pigmentation in different colored crucian carp. Further biochemical and physiological research on these candidate marker genes should be implemented in the future.

## Methods

### Material

RCC and WCC, 1 year old, were collected from the Engineering Center for Fish Breeding of the National Education Ministry, Hunan Normal University. Pieces of skin tissue were surgically excised from RCC and WCC, being anesthetized with 100 mg/L MS-222 (Sigma-Aldrich) before dissection. Each sample of skin tissue was collected from siblings, a mixture of six fishes, which were subsequently used for RNA and DNA isolation. All the sampling procedures were conducted according to the standards and ethical guidelines established by the Animal Ethical Review Committee of Hunan Normal University, Changsha, China.

### cDNA library construction and sequencing

Total RNA was isolated from skin tissues using Trizol Reagent (Invitrogen) following the manufacturer’s protocol. After removing genomic DNA using DNase I (Fermentas, Vilnius, Lithuania), cDNA was constructed from 2 μg of total RNA per sample following the protocol for the Illumina HiSeqTM 2500 (Illumina Corp., San Diego, CA, USA). Total RNA was purified using RNA Trizol (Invitrogen) and quantified with the Agilent 2100 Bioanalyzer (Agilent, Santa Clara, CA, USA). The cDNA libraries were synthesized using the mixed mRNA fragments as templates and the TruSeq RNA sample preparation kit V2 (Illumina), according to the manufacturer’s instructions. The libraries were sequenced using the Illumina HiSeq 2500 at the BGI Shenzhen, Guangdong, China.

### De novo assembly and functional annotation of Transcriptome

Raw reads were filtered using FastQC software to obtain the paired-end clean reads, and all clean reads were used for assembly with Trinity [40, 41]. The assembled transcripts were annotated by BLASTx (National Center for Biotechnology Information [NCBI], Bethesda, MD, USA) against the protein databases of the NCBI, nonredundant (Nr), Swiss-Prot, Kyoto Encyclopedia of Genes and Genomes (KEGG), Clusters of Orthologous Groups (COG), and Gene Ontology (GO) with an e-value of 1e^-5^. GO annotation was performed according to the Nr annotation using Blast2GO software [42], and the GO functional classification was classified using WEGO software [43]. The relevant biological pathways were identified through gene enrichment analyses of the KEGG categories and annotation [44]. The number of genes included in each KEGG pathway was counted and the significance of gene enrichment for each KEGG pathway was calculated using the hypergeometric distribution test.

### Levels of transcript expression and detection of differentially expressed transcripts

CD-HIT software was used to cluster transcripts and remove redundant sequences in transcriptomes of the two crucian carp strains and the referenced sequence data obtained [45]. Then, the numbers of fragments that mapped to each reference transcript were calculated and BLAT was used to align the paired-end reads to these reference sequences in each transcriptome [46]. The transcript expression level was calculated using the fragments per kilobase per million fragments mapped (FPKM) method [47]. The reference transcripts were filtered out when FPKM <0.07 in all samples of each group. Unigenes with FDR ≤ 0.001 and |log2Ratio| ≥ 2 were considered the DEGs for further analysis.

### Quantitative real-time polymerase chain reaction (PCR) analysis

The real-time PCR analysis [48] was performed using the Prism 7500 Sequence Detection System (Applied Biosystems, Foster City, CA, USA) with a miScript SYBR Green PCR kit (Qiagen, Valencia, CA, USA). The amplification conditions were as follows: 50 °C for 5 min and 95 °C for 10 min, followed by 40 cycles at 95 °C for 15 s and 60°Cfor 45 s. The average threshold cycle (Ct) was calculated for each sample using the 2^-∆∆Ct^ method, and normalized to β-actin. Finally, a melting curve analysis was completed to validate the specific generation of the expected product. Primers are available in Additional file 1: Table S3. For each sample, the qRT-PCR analysis was performed by three biological replicates.

### DNA sample isolation and MethylRAD library preparation and sequencing

Genomic DNA of the two crucian carp strains was extracted from skin tissues using the conventional cetyltrimethyl ammonium bromide method following the manufacturer’s protocol. The MethylRAD library was prepared by digesting genomic DNA using FspEI (New England Biolabs, Ipswich, MA, USA) at 37 °C for 4 h. Then, the ligated products were amplified in 20 μl reactions. PCR products were purified using a QIAquick PCR purification kit (Qiagen) and were subjected to single end sequencing on an Illumina HiSeq2500 sequencer. The methyl-RAD methylated tag libraries were constructed using the MethylRAD technique and sequenced on the Hiseq X Ten platform. Raw reads were first subject to quality filtering and adaptor trimming. The FspEI sites (CmC/mCDS) extracted from the crucian carp genome were built as reference sites, and high-quality reads were mapped against these reference sites using the SOAP program. The number of reads supporting methylcytosines was calculated to quantifythe MethylRAD data and normalized as RPM (RPM = (read coverage per site/high-quality reads per library) × 1,000,000).

### DNA methylation data analysis

Raw reads were trimmed to remove adaptor sequences as well as the terminal 2-bp length from each site to eliminate artifacts. The MethylRAD data were analyzed using reference-based and de novo approaches. The Methyl-RAD sequencing tag for each sample was compared to the reference genome (Reference genome download link: https://ftp.ncbi.nlm.nih.gov/Genomes/all/GCF./000/951/615/GCF_000951615.1_common_carp_genome/GCF_000951615.1_common_carp_genome_genomic.fna.gz) using the SOAP software (ver. 2.21; the primary alignment parameters included: -r 0, −M 4). DNA methylation sites with a sequence depth of no less than 3 were judged to be reliable. The function of the gene was described by a GO function enrichment analysis of the gene where the differential methylation site was located. The number of genes included in each GO entry was counted and the significance of gene enrichment for each GO entry was calculated using the hypergeometric distribution test [49].

### Western blotting

We analyzed conservation of some proteins encoded by the DEGs and selected microphthalmia associated transcription factor a (MITFa), tyrosinase protein (TYR), and the dopachrome tautomerase protein (DCT), for which commercially available antibodies were available for western blotting. First, samples containing 50 μg of total lysed protein from RCC and WCC skin tissues was separated by15% sodium dodecyl sulfate-polyacrylamide gel electrophoresis for 1–2 h at 100 V, transferredto a polyvinylidene fluoride membrane (GE Healthcare Bio-Sciences, Uppsala, Sweden), and blocked in TBST containing 5% nonfat milk for 1 h at room temperature. The membrane was incubated with primary antibodies (rabbit polyclonal antibody to MITFa, TYR and DCT, 1:2000; GeneTex Inc., Irvine, CA, USA) overnight at 4 °C. The blots were then washed extensively and incubated with the corresponding peroxidase-conjugated anti-rabbit IgG (1:2000, Sigma St. Louis, MO, USA) for 1 h at room temperature. Proteins were detected using an enhanced chemiluminescence kit (Thermo Fisher Pierce, Rockford, IL, USA) and the ChemiDoc XRS+ imaging system (Bio-Rad, Hercules, CA, USA).

## Additional file

Additional file 1: Table S1. Statistics of function annotation of Red and White skin crucian carp. **Table S2.** Sample sequencing data volume and comparison rate. **Table S3.** List of primers for the qRT-PCR validation of Unigenes identified. **Figure S1.** The GO function is classified by histogram of differences in methylation sites. (DOCX 107 kb)

## Abbreviations

COG: Clusters of Orthologous Groups
DCT: Dopachrome tautomerase
DEGs: Differentially expressed genes qRT-PCR:quantitative polymerase chain reaction
GO: Gene Ontology
HPDA: 4-hydroxyphenylpyruvate dioxygenase
KEGG: Kyoto Encyclopedia of Genes and Genomes
MAPK: Mitogen activated protein kinase
MITFa: Microphthalmia associated transcription factor a
NCBI: National Center for Biotechnology Information
RCC: Red crucian carp
TYR: Tyrosinase
TYRP1: Tyrosinase-related protein 1
VEGF: vascular endothelial growth factor
WCC: White crucian carp
