# Supplementary material for: Comparative Transcriptome and DNA methylation analyses of the molecular mechanisms underlying skin color variations in Crucian carp (Carassius carassius L.)
Source: BMC Genet. 2017 Nov 9;18:95. doi: 10.1186/s12863-017-0564-9 (PMC5680753; doi:10.1186/s12863-017-0564-9)
Supplement: Additional file 1: Table S1. — Statistics of function annotation of Red and White skin crucian carp. Table S2. Sample sequencing data volume and comparison rate. Table S3. List of primers for the qRT-PCR validation of Unigenes identified. Figure S1. The GO function is classified by histogram of differences in methylation sites. (DOCX 107 kb) [file 12863_2017_564_MOESM1_ESM.docx]

**Supplemental Materials**

**Supplemental Table 1. Statistics of function annotation of Red and White skin crucian carp**

| Annotated database | Annotated number | Percentage |
| --- | --- | --- |
| NR | 42,619 | 74.12% |
| NT | 53,950 | 93.83% |
| Swiss-Prot | 40,063 | 69.68% |
| COG | 16,804 | 29.23% |
| GO | 24,971 | 43.43% |
| KEGG | 34,193 | 59.47% |
| All annotated Unigenes | 54,654 | 95.07% |
| All-Unigene | 57,494 | 100% |

**Supplemental Table 2. Sample sequencing data volume and comparison rate**

| Sample | CleanData | Methyl-RAD label | Data(bp) | Logarithm | Match rate |
| --- | --- | --- | --- | --- | --- |
| WCC | 145,718,466 | 16,568,891 | 528,814,258 | 2,995,789 | 18.08% |
| RCC | 145,718,466 | 17,147,966 | 547,677,806 | 3,678,899 | 21.45% |
| Average | 145,718,466 | 16,858,429 | 538,246,032 | 3,337,344 | 19.77% |

**Supplemental Table 3. List of primers for the qRT-PCR validation of Unigenes identified**

| Gene | Primers | sequences(5'to3') | Product Size(bps) |
| --- | --- | --- | --- |
| **Tyr** | Tyr+ | TCCTTCAGTTCTTGAGTCCG | 248 |
|  | Tyr- | TCCTTCAGTTCTTGAGTCCG |  |
| **Tryp1** | Tryp1+ | GGCCACCTATCAGAAACGCT | 240 |
|  | Tryp1- | ATCCTCTGAGTAGCGCCTG |  |
| **Dct** | Dct+ | GAGACACGCTTCTGGGTCC | 239 |
|  | Dct- | GATTCGGGATGGGTCACTGG |  |
| **Mitfa** | Mitfa+ | TATGGCCCTTCTCACCCTC | 220 |
|  | Mitfa- | CCCGGAGATGGAGTAACGG |  |
| **Sox10** | Sox10+ | CCCGAGTACAAGTACCAGCC | 249 |
|  | Sox10- | TTTTCCTCCCTGCAGTTCCG |  |
| **Wnt5b** | Wnt5b+ | GGATGTGAGAATGAACCAAG | 277 |
|  | Wnt5b- | TTGCCTGAACTGAAACTGAC |  |
| **Wnt2b** | Wnt2b+ | TCAAGTTCGCCAAAGCCT | 270 |
|  | Wnt2b- | CTGTGAATCCTGTCCCATC |  |
| **Gsk3β** | Gsk3β+ | AAGTCAGCAGAGATAAAGATGG | 262 |
|  | Gsk3β- | TAACGCAGACGCACAATG |  |
| **Foxd3** | Foxd3+ | CGGACATTCTCAGGGACCAG | 274 |
|  | Foxd3- | TTGATAATCGACGCGGTGCT |  |
| **Hpda** | Hpda+ | TGTGGCTTTTCAAGTGGA | 238 |
|  | Hpda- | CAGTTTTGCTAACAGAGGGT |  |
| **C-myc** | C-myc+ | TAACGCAGACGCACAATG | 263 |
|  | C-myc- | ATCACTTTCCATTTGACCAG |  |
| **Braf** | Braf+ | AATCCTCATCGTCGTTGTC | 265 |
|  | Braf- | GGGAGAAGAAGCCCATTG |  |
| **Hsp70** | Hsp70+ | CAGTTTTGCTAACAGAGGGT | 292 |
|  | Hsp70- | CTGTGTGGGGGTGTTTCA |  |
| **Atg13** | Atg13+ | GTCGTAGATGGAGATGTTGC | 255 |
|  | Atg13- | ATCTTCATCACCGACAGGG |  |
| **Atg9a** | Atg9a+ | TTGAGCAGCAGAGACAGGC | 269 |
|  | Atg9a- | ACTGATTCCTCAAAGGTTACG |  |
| **Tsc1a** | Tsc1a+ | TGCTCCTTCTGGATCTCAAT | 229 |
|  | Tsc1a- | CCCACCTCTCAGGACGAAT |  |
| **Gch1** | Gch1+ | GGAATCTTGGCATCACTTG | 285 |
|  | Gch1- | GGGCTCCTCAAAACTCC |  |
| **Ptps** | Ptps+ | CACCTCGCATAACCATACA | 231 |
|  | Ptps- | AGACCAGACACCAGCAT |  |
| **Sox9b** | Sox9b+ | CGACATCAGCAAAATACG | 281 |
|  | Sox9b- | GACGAGGAGGGGGACAAGTT |  |
| **β-actin** | Actin+ | CAGGGAAAAGATGACACAG | 200 |
|  | Actin- | GTCCATCACGATACCAGT |  |


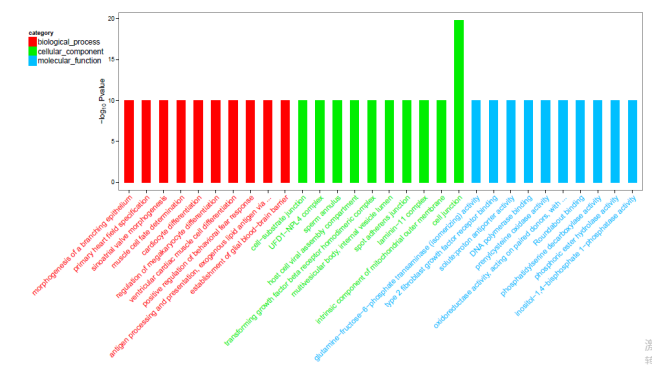


**Supplemental Fig.1 The GO function is classified by histogram of differences in methylation sites.**
